# Predictive Factors for COVID-19 Severity in Patients with Axial Spondyloarthritis: Real-World Data from the Romanian Registry of Rheumatic Diseases

**DOI:** 10.3390/medicina61030411

**Published:** 2025-02-26

**Authors:** Andreea-Iulia Vlădulescu-Trandafir, Violeta-Claudia Bojincă, Cristina Popescu, Constantin Munteanu, Andra-Rodica Bălănescu, Aurelian Anghelescu, Justin Aurelian, Roxana Bistriceanu, Sebastian Giuvara, Elena Grădinaru, Emanuela-Elena Mihai, Daniel Nițu, Mihaela-Ruxandra Vintilă, Gelu Onose

**Affiliations:** 1 Faculty of Medicine, University of Medicine and Pharmacy “Carol Davila”, 020022 Bucharest, Romania; andreea-iulia.trandafir@drd.umfcd.ro (A.-I.V.-T.); violeta.bojinca@umfcd.ro (V.-C.B.); andra.balanescu@umfcd.ro (A.-R.B.); roxana.bistriceanu@drd.umfcd.ro (R.B.); emanuela-elena.mihai@drd.umfcd.ro (E.-E.M.); mihaela-ruxandra.udrea@drd.umfcd.ro (M.-R.V.); gelu.onose@umfcd.ro (G.O.); 2 Neuromuscular Rehabilitation Clinic Division, Teaching Emergency Hospital “Bagdasar-Arseni”, 041915 Bucharest, Romania; aurelian.anghelescu@umfcd.ro (A.A.); sebastian.giuvara@gmail.com (S.G.); 3 Internal Medicine and Rheumatology Departments, “Sfânta Maria” Hospital, 011172 Bucharest, Romania; elena.gradinaru@rez.umfcd.ro (E.G.); nitu.daniel95@yahoo.com (D.N.); 4 Faculty of Medical Bioengineering, University of Medicine and Pharmacy “Grigore T. Popa” Iasi, 700454 Iasi, Romania; 5 Faculty of Midwifery and Nursing, University of Medicine and Pharmacy “Carol Davila”, 020022 Bucharest, Romania; justin.aurelian@umfcd.ro; 6 Department of Urology, “Prof. Dr. Th. Burghele” Clinical Hospital, 050653 Bucharest, Romania; 7 Department of Allergology and Clinical Immunology, “Carol Davila” Nephrology Clinical Hospital, 010731 Bucharest, Romania

**Keywords:** axial spondyloarthritis, coronavirus disease, SARS-CoV-2, RRBR, health literacy, cohort study

## Abstract

*Background and Objectives*: Coronavirus disease-2019 (COVID-19) posed unique challenges worldwide, underscoring important gaps in healthcare preparedness for patients receiving immunosuppressive therapies, such as the individuals with axial spondyloarthritis (axSpA), a subgroup of spondyloarthritis (SpA) characterized by chronic inflammation and immune dysregulation. While global registry data exist for SpA, specific data on axSpA alone remain scarce, especially in Central and Eastern European populations. This study aims to identify predictive factors for severe COVID-19 outcomes and provide a descriptive analysis of axSpA patients infected with the severe acute respiratory syndrome coronavirus 2 (SARS-CoV-2), using real-world data from the Romanian Registry of Rheumatic Diseases (RRBR). *Materials and Methods*: This is a three-year retrospective observational cohort study that included 5.786 axSpA patients from the RRBR, of whom 183 (3.16%) were diagnosed with SARS-CoV-2 infection. Data were analyzed using R V4.4.1 and performing univariate and multivariate binary logistic regression to estimate associations using odds ratios (ORs), 95% confidence intervals (CIs), and *p*-values. A backward selection algorithm was applied to create the final predictive model, accounting for multicollinearity through variance inflation factors (VIFs). *Results*: The mean age of patients was 48.19 ± 12.26 years, with male predominance (64.5%). Serious COVID-19 (encompassing moderate to critical cases) occurred in 46 cases, with age ≥ 52.5 years (OR 2.64, 95% CI: 1.28–5.48, *p* = 0.009) and arterial hypertension (OR 2.57, 95% CI: 1.29–5.16, *p* = 0.007) identified as significant predictors. Individuals with advanced education levels had nearly three times lower odds of experiencing serious COVID-19 (OR 0.38, 95% CI: 0.18–0.76, *p* = 0.008). Furthermore, our findings confirm the lack of association between HLA-B27 and COVID-19 severity (*p* = 0.194), contributing to the ongoing discussion regarding its potential immunological role. Moreover, irrespective of the biological therapy administered, the likelihood of experiencing serious SARS-CoV-2 outcomes was not statistically significant (*p* = 0.882). In the final predictive model, only older age and higher education were deemed as predictive factors. *Conclusions*: This study highlights key predictors of COVID-19 severity in axSpA patients and emphasizes the protective role of higher education, an underexplored determinant of health outcomes in inflammatory diseases. The lessons learned during these last years can shape a more informed and compassionate healthcare system.

## 1. Introduction

The novel coronavirus infection that determined the seventh pandemic recognized globally in the last 130 years rapidly escalated into an important public health problem [1,2,3]. The tremendous strain that it brought caused widespread physical and psychological repercussions as many healthcare systems were struggling and fighting for better solutions [1,3,4]. As of 30 November 2024, more than 700 million cases (including reinfections) and over 7 million fatalities have been recorded worldwide [5].

The COVID-19 pandemic underscored serious gaps in healthcare preparedness for patients receiving immunosuppressive drugs, including biologic therapy. Concerns were raised amid individuals suffering from immune-mediated and inflammatory rheumatic diseases (IMIDs), prompting increased scientific efforts to assess their vulnerability to SARS-CoV-2 [6,7,8]. However, even though the pandemic has officially ended, new cases are still present, and not all the factors interfering with the course of COVID-19 in patients with IMIDs are fully understood, particularly for those with axSpA. Identifying individuals at high risk of severe infections continues to be a priority.

Axial spondyloarthritis is a chronic inflammatory disease characterized by a significant degree of polymorphism [9,10]. It primarily affects the sacroiliac joints and the axial skeleton. However, it may also present with extra-musculoskeletal symptoms, including uveitis, psoriasis, inflammatory bowel disease (IBD), and systemic involvement (pulmonary, cardiac, renal, neurological) [11,12,13]. Additionally, axSpA is associated with multiple comorbidities, some of which are associated with worse COVID-19 outcomes [14,15,16,17].

Due to pandemic-related restrictions, mental health issues, new conditions, and exacerbations of previously existing maladies likely emerged since in-person healthcare consultations were significantly limited, and not all the patients requiring assistance had access to telemedicine or e-health consultations [7,8,18,19,20]. An estimated 58.4% of spondyloarthritis (SpA) patients experienced canceled rheumatology visits, and 45.6% lacked information on the pandemic’s impact on their disease, leading to worsened disease management [17]. The aforementioned trend was similarly observed in various countries and has emerged as a significant public health challenge due to diminished access to healthcare services, pronounced health inequities, reduced quality of life (QoL), and the emergence of new-onset comorbidities that might have gone unaddressed [18,21,22].

It is estimated that more than 90% heritability is involved in the pathogenesis of axSpA, and HLA-B27 is the strongest genetic association. The pathogenic role of HLA-B27 is yet to be determined despite different hypotheses [23,24,25]. Emerging data highlighted the protective character of HLA-B27 in specific viral infections concerning human immunodeficiency virus (HIV), Epstein–Barr, influenza, and herpes simplex 2 viruses; this hypothesis was a catalyst for studies regarding the protective role of this gene for patients with COVID-19 [24,26,27,28].

Additionally, growing evidence indicates that the cytokines tumor necrosis factor-alpha (TNF-α) and interleukin-17 (IL-17) are important in axSpA pathogenesis, especially regarding structural damage and synovial and entheseal inflammation [17]. Biological therapies that inhibit the aforementioned cytokines and the newly approved Janus kinase inhibitors (JAKi) mark an important step forward in slowing the progression of axSpA and preventing articular and extra-musculoskeletal flares [29]. Given that the cytokine storm contributes to COVID-19 severity and mortality [1,2,7,30], early studies explored cytokine inhibitors as potential treatments. However, results were inconsistent, partly due to concerns about reactivating latent infections [6,31,32,33,34,35].

Initiated in 2013, the Romanian Registry of Rheumatic Diseases [36] is a collaborative effort held by Romanian rheumatologists in the form of a national prospective cohort that includes patients with IMIDs treated with reimbursed biologic disease-modifying antirheumatic drugs (bDMARDs) and targeted synthetic disease-modifying antirheumatic drugs (tsDMARDs). The subjects are enrolled at the time of starting either a bDMARD or a tsDMARD therapy in accordance with the national guidelines. The electronic registry is regularly updated by rheumatologists every 6 months, or more frequently if necessary, including additional data on adverse events. The patients are being followed up prospectively until the treatment is discontinued [37,38,39,40].

The aim of the present study was to draw attention to and give insight into the risk factors influencing COVID-19 severity in axSpA patients. Although the pandemic has ended and the number of SARS-CoV-2 cases has reduced significantly, studying COVID-19 severity in individuals with axSpA aids in optimizing care for this specific population and contributes to broader efforts to understand the interaction between chronic inflammatory ailments and infectious diseases.

In a narrative review conducted by Deodhar et al. [17] regarding the impact of COVID-19 on SpA patients (including those with axSpA), the evidence does not indicate an increased risk of severe SARS-CoV-2 outcomes, such as hospitalization and mortality in SpA patients receiving DMARD therapy. This finding is consistent with a separate longitudinal study conducted early in the pandemic, which included 2157 SpA patients from the United States (U.S.) [41]. Moreover, this latter study demonstrated that the incidence rate among U.S. SpA patients was slightly higher than in the general population, the difference being only marginally significant (*p* = 0.06). Similarly, an analysis of 450 household members did not demonstrate a statistically significant increase in COVID-19 risk among SpA patients compared to their household controls [41].

An analysis of data from various registry-based and national cohort studies suggests that patients with axSpA, particularly those receiving TNF-α inhibitor monotherapy, do not face an increased risk of hospitalization or mortality due to COVID-19 [17,42,43,44,45,46]. However, these studies primarily examined broader populations, either including multiple IMIDs or SpA as a whole, rather than focusing specifically on axSpA. In contrast, a matched cohort study from British Columbia, Canada, which analyzed 104,508 COVID-19 cases, identified a significantly higher risk of hospitalization (OR: 2.16) and intensive care unit (ICU) admission (OR: 2.29) among the 378 individuals with axSpA. The study did not report an increased mortality risk in this patient population [47].

Previous studies investigating SpA and COVID-19 often examined heterogeneous populations, including psoriatic arthritis and peripheral spondyloarthritis, making it difficult to draw specific conclusions regarding axSpA alone. Moreover, most data originate from Western European [11,22,32,45,46,48] and North American cohorts [27,41,49], leaving significant gaps in understanding COVID-19 outcomes in Central and Eastern European populations, where healthcare access, treatment patterns, and genetic predispositions may differ. Given these limitations, we sought to investigate the predictors of COVID-19 severity, specifically in axSpA patients, using real-world data from the National Romanian Registry.

The primary objective of the current study was to identify predictive factors associated with the severity of COVID-19 in Romanian patients diagnosed with axSpA. The secondary objective involved performing a descriptive analysis of this specific patient group in relation to their demographics, clinical characteristics, and paraclinical data.

## 2. Materials and Methods

This study presents a retrospective observational cohort study involving 5786 patients from the RRBR diagnosed with axSpA. Among this cohort, 183 individuals (3.16%) were identified as having contracted COVID-19 during the period from 1 March 2020 to 31 December 2023. This number is representative of the patients with IMIDs from the RRBR in Romania who were infected with SARS-CoV-2 [39]. The study closely adheres to the Strengthening the Reporting of Observational Studies in Epidemiology (STROBE) guidelines (Supplementary Material S1).

### 2.1. Ethical Considerations

This study was conducted according to the Declaration of Helsinki, local regulations, and Good Clinical Practice (GCP). Written informed consent, including General Data Protection Regulation (GDPR) authorization, was obtained from all enrolled patients before the study procedures, including using electronic data for medical research purposes when they were first enrolled in the RRBR. Patient personal data have been coded.

### 2.2. Data Sources

We analyzed anonymized data from patients with axSpA and COVID-19 that were prospectively collected in the RRBR at the initiation of a b/tsDMARD therapy. The data were uploaded to a secure server and voluntarily entered into the system by rheumatologists. Data pertaining to adverse effects and complications is typically updated every six months, or more frequently if the circumstances necessitate such action. In our study, the individual who collected and anonymized the raw data did not participate in the statistical analysis phase to minimize potential biases. Subsequently, the data were harmonized, underwent rigorous quality checks, and consolidated before statistical analyses were implemented.

### 2.3. Eligibility Criteria

The study included all patients aged 18 years or older diagnosed with axSpA and SARS-CoV-2 and enrolled in the RRBR. COVID-19 diagnosis was confirmed via a Rapid Antigen or a reverse transcription polymerase chain reaction (RT-PCR) test. All participants had received at least one biological treatment during their inflammatory disease and could provide informed consent.

According to the National Protocol [50], a rheumatologist establishes an axSpA diagnosis based on sacroiliitis confirmation via imaging (magnetic resonance imaging (MRI) with signs of active inflammation or radiograph), accompanied, according to the classification criteria for axSpA, by at least one of the characteristic features: arthritis, heel enthesitis, uveitis, dactylitis, psoriasis, Crohn’s disease/ulcerative colitis, nonsteroidal anti-inflammatory drug (NSAID) response, family history of axSpA, HLA-B27 positivity, or elevated C-reactive protein (CRP) levels. If the patient shows radiographic changes of sacroiliitis meeting the modified New York criteria (1984), the case qualifies as axSpA based on the following:

- Lower-back pain and stiffness lasting for more than three months, improving with exercise and not relieved by rest;
- Limitation of lumbar spine motion in both the sagittal and frontal planes;
- Reduced chest expansion;
- Imaging criterion: unilateral sacroiliitis grade 3–4 or bilateral sacroiliitis grade 2–4 on radiographs.

A definitive diagnosis of axSpA requires the presence of imaging confirmation combined with at least one clinical criterion.

Patients were not considered eligible for the study if they were under 18 years of age, had psychiatric conditions that interfered with the ability to provide informed consent, or had an uncertain COVID-19 diagnostic status.

### 2.4. Variables

Demographic variables included sex, age, smoking status, residence area, and educational level.

The clinical variables were comorbidities and axSpA characteristics, such as disease duration and extra-musculoskeletal manifestations: ocular, pulmonary, cardiological, dermatological, and gastrointestinal. Regarding axSpA treatments, data collected included molecule type and treatment decisions (initiations, continuations, switching), specifically during and after the infection. The following data were collected pertaining to SARS-CoV-2 infection: duration of the disease, the course of the b/tsDMARD therapy used, the clinical manifestations of COVID-19, the severity of the infection, and particular comments that the attending physician wanted to report.

The paraclinical variable was HLA-B27 status. Due to data protection regulations outlined in the Informed Consent Form, we did not have access to laboratory results for SARS-CoV-2 infection or other supplementary data requiring direct patient contact.

### 2.5. Outcome Measures

The primary outcome was to identify predictive factors associated with the severity of COVID-19 in patients diagnosed with axSpA. We described the term “serious COVID-19” to confine moderate, severe, or critical cases that generally require targeted medical interventions. In addition, the secondary outcome was identifying the specific axSpA phenotype that was susceptible to SARS-CoV-2 infection.

### 2.6. Statistical Analysis

The data were analyzed using R V4.4.1. Copyright (C) 2024, The R Foundation for Statistical Computing, R Core Team (2024). R: A language and environment for statistical computing. R Foundation for Statistical Computing, Vienna, Austria. URL (https://www.R-project.org, accessed on 18 December 2024) [51]. Descriptive statistics were computed for demographic and patient characteristics, including mean, standard deviation (SD), median, interquartile range, and percentages.

Different univariate and multivariate binary logistic regression analyses were conducted to identify predictive factors for a “serious” SARS-CoV-2 progression. The dependent variable was classified as either the presence or absence of a serious disease course. The independent variables comprised demographic, clinical, and paraclinical parameters that were monitored throughout the study. Associations were estimated using OR, 95% CI, and *p*-values. The α significance threshold was considered at 0.05 and *p* < 0.05 as having statistical significance.

A backward selection algorithm was applied to establish the final predictive model, considering multicollinearity by VIF. Values larger than the commonly accepted threshold (e.g., VIF > 5) indicate problematic correlations that could distort the results. The final model achieved an optimal balance between complexity and explanatory power, retaining only predictors that demonstrated statistical significance and clinical relevance while excluding redundant or collinear variables.

## 3. Results

### 3.1. Demographics

The study cohort included 5.786 patients diagnosed with axSpA based on the National Protocol criteria [50], as detailed in the Section 2, that have been enrolled in the RRBR and of whom 183 patients (3.16%) were also diagnosed as having COVID-19. The infection was diagnosed via Rapid Antigen or RT-PCR testing. Among the assessed individuals, 65 (35.5%) were female, and 118 (64.5%) were male. This distribution highlights the increasing awareness of this diagnosis within the female population, challenging the historical perception of axSpA as predominantly a male disease [10,52].

The mean age at the time of COVID-19 diagnosis was 48.19 ± 12.26 years, with no significant sex-based differences (females: 48.32 ± 14.12 years; males: 48.12 ± 11.17 years).

Pertaining to the area of residence, a significant majority, totaling 138 individuals (75.4%), resided in urban areas. In contrast, the remaining 45 patients (24.6%) lived in rural areas of the country. Surprisingly, the percentage of females in both rural and urban settlements was identical, with each area reporting a percentage of 35.5%. The remaining patients in both residence groups were male, maintaining sex balance across locations. The sex distribution in both residence groups mirrored the overall cohort, with 35.5% being female.

The distribution of educational levels revealed that 96 individuals (52.5%) had an elementary or high school education, while 87 patients (47.5%) had completed higher education, defined as at least a university degree.

Regarding smoking status, 27 individuals (14.8%) were current or former smokers, with a mean smoking duration of 15.29 years, while 156 (85.2%) were non-smokers.

A summary of the data presented is presented in Table 1.

### 3.2. axSpA-Specific Characteristics

Among the study population, we identified 118 patients (64.5%) with HLA-B27 positivity. This prevalence aligns with the established role of HLA-B27 in the pathogenesis of the disease, particularly in promoting inflammation and extra-articular manifestations [23,24,27,53].

**Figure 1:**
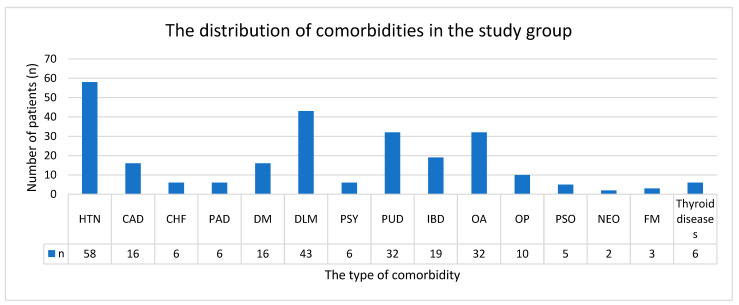
Clustered column chart illustrating the distribution of comorbidities. CAD: coronary artery disease; CHF: congestive heart failure; DLM: dyslipidemia; DM: diabetes mellitus; FM: fibromyalgia; HTN: arterial hypertension; IBD: inflammatory bowel disease; NEO: neoplasia (any type); OA: osteoarthritis; OP: osteoporosis; PAD: peripheral artery disease; PSO: psoriasis; PSY: psychiatric diseases; PUD: peptic ulcer disease.

The burden of comorbidities was significant within the cohort, with a mean of 1.68 ± 1.90 comorbidities per patient. Notably, 119 patients (65%) presented with at least one comorbidity, highlighting the complex clinical profile of axSpA. The most frequently reported comorbid conditions included cardiovascular diseases (37.7%), gastrointestinal diseases (26.2%), dyslipidemia (23.5%), diabetes mellitus (8.7%), and neurological and psychiatric diseases (4.91%). The detailed comorbidities are presented in Figure 1.

The mean duration of axSpA before SARS-CoV-2 infection was 14.6 ± 8.72 years, highlighting the chronic nature of the disease and its prolonged inflammatory burden that may influence disease outcomes and comorbidity profiles.

Extra-musculoskeletal manifestations are hallmark features of axSpA, reflecting the systemic involvement of the disease beyond the musculoskeletal system. In this cohort, such manifestations were identified in several organ systems.

Anterior uveitis and other ocular manifestations were reported in 54 patients (29.5%), consistent with its well-established association with axSpA. According to meta-analyses, the prevalence of anterior uveitis in spondyloarthritis ranges from 21 to 33% [13,54]. Pulmonary involvement, such as interstitial lung disease, apical fibrosis, emphysema, and bronchiectasis, was recorded in 17 patients (9.3%). Gastrointestinal manifestations, found in four cases (2.2%), indicate overlapping inflammatory conditions such as Crohn’s disease or ulcerative colitis. Dermatological involvement was recorded in five individuals (2.7%), reflecting associated psoriasis or psoriatic-like conditions. Neurological manifestations were rare, occurring in only one patient (0.5%). Cardiac involvement, particularly conduction disturbances and structural changes such as aortic root dilatation and aortic insufficiency, was identified in two cases (1.1%). Renal involvement was present in two individuals (1.1%), which could include complications such as Immunoglobulin A (IgA) nephropathy, or renal amyloidosis.

### 3.3. axSpA Treatments

Cytokines are regulatory proteins that play an important role in the development and progression of inflammatory diseases. Among the most extensively studied cytokines are TNF-α and IL-17, which have attracted significant interest due to their incrimination in the pathogenesis of various IMIDs, including axSpA. The development of targeted cytokine therapies represents a meaningful advancement in axSpA management, providing patients with the potential for better health outcomes, including reducing intestinal inflammation [13,55].

In the study cohort, 21 subjects (11.5%) were undergoing treatment with IL-17 inhibitors, specifically Secukinumab. However, the majority, 162 individuals (88.5%), were treated with TNF-α inhibitors.

In addition to biological therapies, a subset of patients received conventional synthetic disease-modifying antirheumatic drugs (csDMARDs): Methotrexate (MTX), eight patients (4.4%); Sulfasalazine (SSZ), thirteen patients (7.1%). Available data so far suggest that csDMARDs (especially MTX) and anti-TNF-α comedication therapy, compared with anti-TNF-α monotherapy, are not superior in terms of treatment response but may be beneficial for biological treatment retention by reducing antidrug antibodies in axSpA and also in psoriatic arthritis (PsA) [56,57].

#### Treatment Dynamics

Regarding the management of biologic therapies during SARS-CoV-2 infection, 31 (16.9%) patients maintained uninterrupted treatment, while 131 temporarily suspended therapy, typically for a short duration of 2–3 weeks during the course of the infection. This decision was made by the attending physician on a case-by-case basis. Additionally, 21 individuals permanently discontinued treatment, presumably transitioning to alternative therapeutic regimens. It is important to note that in clinical practice, patients with mild symptoms often reported the infection retrospectively during follow-up visits, which may have precluded adjustments in biologic therapy at the time of infection. Following the temporary suspension, most patients resumed their biological treatment.

When consulting the most recent Registry entry for each patient to see the dynamic of the treatment, we observed that the distribution of the current biological therapy used was as follows: 142 patients continued their biological agent, while 40 switched to a different one. TNF-α inhibitors were the most frequently utilized class (155 patients), followed by IL-17 inhibitors, which were prescribed to 27 individuals. Within the IL-17 inhibitor group, Secukinumab was prescribed to 26 patients and Ixekizumab to 1 patient, reflecting an increase in the utilization of this therapeutic class.

Patients had an average of 1.82 ± 1.07 biologic therapy changes before COVID-19 onset, with some switching up to six biologics, highlighting the challenges in achieving optimal disease control in certain cases. The mean duration of biologic therapy before COVID-19 diagnosis was 3.2 ± 2.94 years, emphasizing the established use of these advanced therapeutic agents in this patient population.

### 3.4. COVID-19 Data

According to the World Health Organization (WHO), the clinical classification of COVID-19 includes asymptomatic, mild, moderate, severe, and critical cases.

Asymptomatic: This category includes individuals who do not exhibit any signs or symptoms of SARS-CoV-2 infection but have tested positive for the virus through either an RT-PCR test or a Rapid Antigen test.

Mild cases include patients who present with COVID-19-related symptoms. However, they do not show any signs of viral pneumonia, and their peripheral capillary oxygen saturation levels (SpO_2_) remain at normal levels. This group generally does not require hospitalization and may be managed with home isolation and supportive care.

Moderate: Individuals classified as moderate have been diagnosed with non-severe pneumonia resulting from COVID-19. They demonstrate a systemic response to the infection but maintain a SpO_2_ greater than 90%, while breathing room air and may require supplemental oxygen or other interventions to prevent further deterioration.

Severe: This classification corresponds to cases of severe pneumonia. Patients typically have important dyspnea and a SpO_2_ < 90% in the ambient air and often require hospitalization for intensive monitoring and management.

Critical: Patients in this category are experiencing life-threatening conditions such as acute respiratory distress syndrome (ARDS), septic shock, or acute thromboembolic events. These individuals require immediate and comprehensive medical care, often in the ICU, where advanced therapeutic measures like mechanical ventilation or other life support systems may be implemented [21].

In light of this classification and to enhance the stratification of infection severity in our cohort, we have delineated two categories: mild COVID-19, which encompasses asymptomatic and mild cases that generally do not necessitate hospitalization, and serious COVID-19, comprising moderate, severe, or critical cases that typically require specialized medical care.

The cohort’s SARS-CoV-2 diagnoses were confirmed through RT-PCR or Rapid Antigen tests, following protocols established by the WHO and Romanian national guidelines [58,59].

The average SARS-CoV-2 symptoms duration was 12.32 ± 6.25 days, suggesting a protracted disease course compared to typical upper respiratory infections. The clinical presentation was dominated by pulmonary symptoms experienced by 168 patients (91.8%), which underlines the virus’s tropism for respiratory tissues. Neurological symptoms occurred in 17 cases (9.3%), ranging from headache and anosmia to more severe manifestations such as confusion and dizziness. Notably, most patients with neurological symptoms presented moderate to severe forms of COVID-19, with only two mild cases, suggesting a possible association with disease severity. Gastrointestinal manifestations, including nausea, diarrhea, and abdominal pain, were rare and occurred in only four patients (2.2%).

### 3.5. COVID-19 Severity

Forty-six (25.1%) cases in our cohort were classified as serious COVID-19. Among them, one progressed to a critical stage necessitating mechanical ventilation and intubation; this patient ultimately succumbed to the disease.

**Figure 2:**
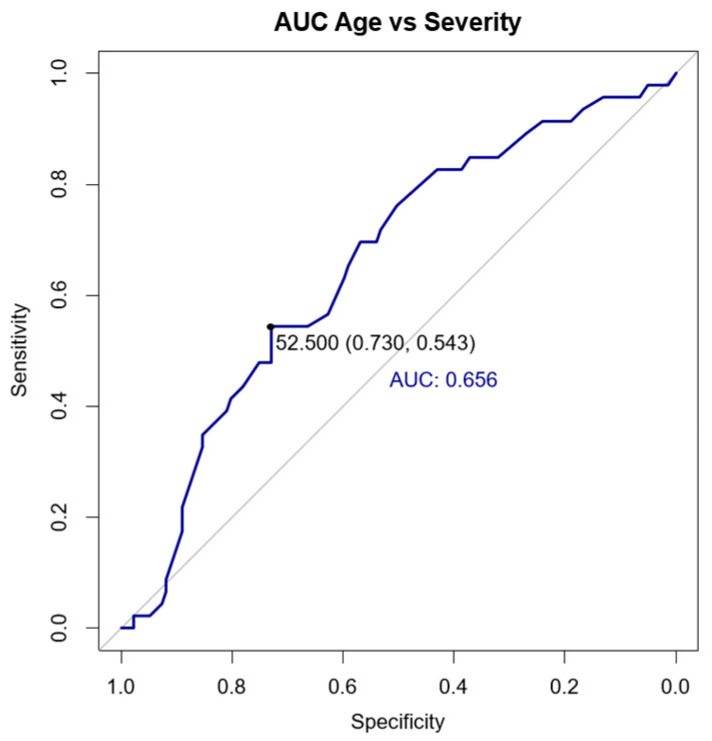
ROC analysis for determining cut-off age in the cohort.

The cut-off age for categorizing patients into age groups was determined using a receiver operating characteristic (ROC) analysis, a statistical method used to identify the optimal threshold that maximizes both sensitivity and specificity for predicting severe disease outcomes (Figure 2). This finding should be interpreted cautiously, given the limited number of serious cases (n = 46) compared to larger global cohort studies.

This analysis suggests that patients older than 52.5 years have a higher likelihood of serious COVID-19, with an area under the curve (AUC) of 0.656. To increase the specificity, we conducted univariate binomial logistic regression analysis of the study variables.

The detailed data for the univariate binomial logistic regression analysis are presented in Table 2.

The current literature identifies male sex as a risk factor associated with the severity of SARS-CoV-2 infection [48,60,61]. In the entire cohort, 65% of the individuals were male. In our analysis, serious COVID-19 was observed in 20 females and 26 males. However, the comparison between sex groups did not demonstrate statistical significance (*p* = 0.194).

On the other hand, a positive association was observed between age and the likelihood of developing serious COVID-19. Specifically, for every additional year of age, there was a corresponding 4% increased odds of serious infection, amplifying the concept of accrual of risk factors across life, probably due to immunosenescence and a higher number of comorbidities in older individuals. Moreover, patients older than 52.5 years had a remarkably higher risk, with an odds ratio of 3.22 for serious SARS-CoV-2 outcomes compared to younger patients (*p* < 0.001).

An interesting observation can be made regarding socioeconomic status: the level of education, but not the living environment, contributes to COVID-19 severity. Higher education (defined as minimum university schooling) was associated with a significant protective effect (*p* = 0.008), as patients with advanced education levels had nearly three times lower odds of experiencing serious disease than those with primary or secondary education.

While smoking is often identified as a risk factor associated with the severity of respiratory infections [62], the prevalence of smokers within our cohort was notably low, at 14.7%. Furthermore, among those classified in the SARS-CoV-2 severity group, only eight cases, equating to 4.37%, were smokers. Regression analysis indicated that smoking was not a significant risk factor in our cohort (*p* = 0.561).

In relation to the utilization of csDMARDs, there were eight patients receiving MTX treatment and thirteen undergoing SSZ treatment, among whom only one and, respectively, five experienced serious COVID-19. These treatments did not significantly impact the SARS-CoV-2 severity (*p* = 0.414 for MTX; *p* = 0.258 for SSZ).

Concerning the biological therapy utilized, we identified five serious cases of COVID-19 within the IL-17 inhibitors cohort, accounting for 23.8% of the total users of this drug. Additionally, there were forty-one serious cases in the anti-TNFα group, representing 25.3% of the overall users in that category. The statistical analysis indicated no significant difference between the two groups (*p* = 0.882). Additionally, the patients had experienced an average of 1.8 changes in biological therapy and had a mean duration of treatment of 3.17 years. This places this group within the middle range of the overall cohort, which had an average of 1.82 ± 1.07 changes in biological therapy and a mean duration of 3.2 ± 2.94 years of treatment before their COVID-19 diagnosis.

Out of the total cohort, 108 patients were vaccinated, of whom 20 developed serious COVID-19, with no statistically significant differences between groups (*p* = 0.470). However, vaccination data were collected solely as a binary “yes/no” response, without information on the type of vaccine administered or the time interval between vaccination and infection, an important potential source of bias.

Regarding comorbidities and COVID-19 severity, we identified arterial hypertension as having statistical significance. HTN was strongly associated with the infection severity, with affected patients having a 2.57-fold increase in the odds of serious disease (*p* = 0.007). Furthermore, individuals with osteoporosis exhibited 3.22 times higher odds of serious disease. Although this association was marginally non-significant (*p* = 0.07), it suggests a possible link worth exploring further.

The presence of HLA-B27 did not provide protective benefits, nor did it increase the risk of serious COVID-19. Among the 46 identified serious SARS-CoV-2 cases, 26 individuals tested positive for this specific gene; however, no statistically significant difference was observed between HLA-B27 carriers and non-carriers regarding the severity of the infection (*p* = 0.194).

#### Predictive Model Development

All the variables with a *p*-value less than 0.10 were selected for inclusion in multiple univariate binomial logistic regression to identify independent contributors of serious disease. This cut-off was chosen to ensure the inclusion of marginally significant variables and not prematurely exclude some predictors that may be important.

The initial model, presented below (Table 3), incorporated these predictors to assess their adjusted effects on severe disease outcomes, accounting for potential confounders.

A final predictive model was identified using a backward selection algorithm based on the *p*-values and VIFs associated with the predictors and is presented in Table 4.

The final model revealed that patients aged 52.5 years or older had 2.64 times higher odds of developing serious COVID-19 than younger individuals. Additionally, patients with higher education had 50% lower odds of experiencing serious infection compared to those with only elementary or high school education. It can be observed that after allowing for age, neither HTN nor osteoporosis are significant predictors of the risk of serious COVID-19.

## 4. Discussion

National registries, such as the RRBR, provide invaluable longitudinal data for patients with axSpA undergoing treatment with reimbursed b/tsDMARDs.

To our knowledge, this is the first national study examining the relationship between axSpA and COVID-19 in RRBR-registered patients. Due to the underrepresentation of Central and Eastern European populations in global rheumatology registries, these findings contribute to a broader understanding of COVID-19 risks in diverse patient groups and healthcare systems. This remains a relevant topic in ongoing discussions about identifying patients at risk for severe pulmonary infections [63]. The primary objective of this research was to identify risk factors associated with the severity of infection using various statistical analyses, including univariate and multivariate binomial regression models and a backward selection algorithm. Additionally, we performed a descriptive analysis of this particular patient group, identifying the specific axSpA phenotype that had an increased susceptibility to SARS-CoV-2 infection. Both objectives were met and are discussed cohesively.

The male predominance (64.5%) in our cohort aligns with the literature data but also points out the growing awareness of axSpA in females, which is beyond previous conventional perceptions of axSpA being considered a predominantly male disease. The changing diagnostic trend likely represents better recognition of non-radiographic and atypical female presentations, as noted in contemporary studies [64,65]. Although male sex is linked to increased SARS-CoV-2 severity [48,60,61,66], in our cohort, there was no statistical difference (*p* = 0.194).

Age was identified as an important non-modifiable predictor. The mean age at COVID-19 diagnosis was 48.19 ± 12.26 years, which aligns with the common age range of active axSpA patients in Romania [37,38]. The age range represented by this population encompasses various age groups, thereby enhancing the statistical analysis. We conducted an ROC analysis to identify a cut-off age, finding that patients older than 52.5 years are more likely to experience serious COVID-19 (AUC: 0.656). Our final model indicates that those aged 52.5 or older have 2.64 times higher odds of serious SARS-CoV-2 infection compared to younger patients (*p* = 0.009). Even though we acknowledge the relatively small number of axSpA patients in this cohort as an important limitation, it is important to emphasize the significant role of age as a predictor of COVID-19 severity, likely reflecting the cumulative effects of age-related physiological changes, immunosenescence, reduced B- and T-cell counts, and metabolic status (mainly of vitamin D multifactorial deficiency). Additionally, the higher prevalence of comorbidities in older adults may further contribute to this increased severity [66,67,68,69]. Our research is consistent with various studies made with bigger cohorts that underscore age as a significant predictor for worse COVID-19 outcomes, either on IMIDs [42,70,71] or SpA patients [46,72,73].

Health literacy has a powerful role in protecting individuals against the severity of COVID-19, according to data from the literature [66,74,75,76]. This observation may reflect the role of education in promoting a simple but efficient ensemble of prophylactic endeavors, improving chronic disease self-management, enhancing treatment adherence, and encouraging timely healthcare-seeking behaviors. Furthermore, patients with higher education are expected to have better knowledge about disease prevention, including adopting precautionary behaviors like mask-wearing and social distancing, as well as having better access to telehealth, being more adaptable, and perceiving the virus as a manageable situation [75,76,77,78].

The distribution of educational levels in our cohort revealed that 96 patients (52.5%) had an elementary or high school education, while 87 (47.5%) had completed higher education, defined as at least a university degree. Following the final analysis model, we determined that higher education serves as a protective factor, reducing the likelihood of experiencing serious COVID-19 outcomes by 50%. While the role of socioeconomic status and health literacy in SARS-CoV-2 infection has been discussed in other studies [66,74,76,77], data specifically linking education levels to COVID-19 severity in axSpA patients remain scarce. These findings highlight the need for targeted patient education strategies, particularly among high-risk populations with lower health literacy.

Smoking is linked to the exacerbation of respiratory infections, including COVID-19; it promotes inflammation by the excess production of TNF-α and transforming growth factor β (TGF-β), and diverse interleukins (IL) such as IL-1β, IL-12, IL-17, and IL-23 also have an impact on gene expression [62,79]. In our cohort, 27 patients (14.8%) were current or former smokers, with an average smoking duration of 15.29 years. In addition, only eight individuals with serious COVID-19 were identified as smokers. Additionally, statistical analysis indicated no significant difference between the smoking and non-smoking groups, yielding a *p*-value of 0.561. The low prevalence of smokers among the study population may partly account for this finding.

Among the study population, 118 patients (64.5%) were positive for HLA-B27, a genetic marker strongly associated with axSpA. This prevalence aligns with other studies from Romania utilizing RRBR data [37,38], as well as international studies [24,27,53], thus making our cohort more representative of axSpA patients. HLA-B27 has an established role in the pathogenesis of axSpA, particularly in promoting inflammation via cytokine production. Its association with the major histocompatibility complex class I makes HLA-B27 directly involved with the endogenous antigen presentation to cytotoxic T cells (T_c_). Additionally, it was demonstrated that this gene promotes a spontaneous T_c_-mediated viral clearance [23,24,27,53]. Many studies have shown that patients with axSpA tend to experience milder forms of COVID-19 compared to those with other rheumatic diseases [32,80], and that HLA-B27 might provide some protection against certain viruses, including HIV, Epstein–Barr, influenza, hepatitis, and herpes simplex type 2 [24,27,28]. However, real-world studies have not confirmed the hypothesis of HLA-B27 as a protective factor for severe COVID-19 outcomes [24,27]. Our study aligns with the literature data, as the positivity for HLA-B27 does not provide protective benefits, nor does it increase the risk of serious SARS-CoV-2 infection (*p* = 0.194). This adds to the ongoing scientific debate regarding the immunomodulatory role of HLA-B27 in viral infections and its clinical relevance in COVID-19.

The substantial comorbidity burden, with 65% of individuals experiencing at least one comorbidity, highlights the multifaceted challenges associated with the management of axSpA patients during a pandemic. However, a notable limitation is the potential underreporting of comorbidities, as patient data in this study were voluntarily provided by attending physicians. Furthermore, the lack of access to personal data precluded direct patient contact or verification with primary care health records.

The existing literature indicates that cardiovascular diseases are particularly concerning due to their potential to worsen COVID-19 outcomes [4,7,21,81,82]. In our cohort, a significant association was identified solely with HTN (in the initial univariate analysis) regarding SARS-CoV-2 severity. Hypertensive patients had a 2.57-fold increase in the likelihood of developing serious outcomes (*p* = 0.007). This is of particular importance for Romanian patients since HTN is the third leading cause of mortality [20].

HTN develops through a multifaceted interaction of biological systems like the sympathetic nervous system, the renin–angiotensin–aldosterone system (RAAS), vascular resistance, and endothelial function, alongside lifestyle factors such as obesity, high salt intake, inactivity, and alcohol use [83,84,85]. SARS-CoV-2 intensifies this risk by impairing Angiotensin-Converting Enzyme 2 (ACE-2), which is ubiquitously distributed in the respiratory system, endothelium, and dopamine-producing neurons in the brain. Moreover, ACE-2 is a crucial RAAS regulator, disrupting the balance between vasoprotective and vasoconstrictive pathways. This imbalance elevates angiotensin II (ANG II) levels, leading to increased vascular tension, arterial stiffness, and higher blood pressure (BP) [30,83,84,86]. Given the increased cardiovascular mortality risk in axSpA patients—estimated at 36–40% higher than in healthy controls—HTN emerges as a critical factor in the broader context of cardiovascular disease management in these populations [86].

Another interesting observation that emerged from the initial univariate analysis was the association of documented osteoporosis with serious COVID-19 cases. Patients with osteoporosis had 3.22 times higher odds of serious infection. Although this association was marginally non-significant (*p* = 0.07), it indicates a possible link worth exploring further. It has to be taken into consideration that the true prevalence of osteoporosis may be underreported since only 10 cases were identified within the total cohort of 183 individuals. Of these ten cases, five had serious COVID-19. Furthermore, after adjusting for age in the final predictive model, osteoporosis was not associated with worse COVID-19 outcomes.

Osteoporosis is a recognized public health concern, with a prevalence of 4.8% in Romanian patients [87,88]. Globally, osteoporosis prevalence in axSpA patients ranges from 8.8% to 34.4%, driven by multifactorial physiopathological mechanisms. Chronic inflammation plays a central role, with elevated pro-inflammatory cytokines, such as TNF-α and IL-6, driving osteoclast activation and bone resorption. Other contributing factors include disease-related immobility, which reduces mechanical loading on bones and accelerates bone loss. Malabsorption syndromes associated with concurrent IBD can impair calcium and vitamin D absorption, worsening bone demineralization [86,89]. Studies suggest that up to 70% of axSpA patients show intestinal inflammation, even without a confirmed IBD diagnosis. This inflammation is predominantly localized within the terminal ileum, where a variety of bacterial species has been identified in significant abundance [55,90].

While direct data on vitamin D levels were unavailable for our cohort (due to cost and lack of routine testing in Romania), numerous studies link its deficiency to severe SARS-CoV-2 outcomes [91,92,93,94,95]. Thus, maintaining adequate levels of vitamin D may constitute a simple yet potentially effective strategy to mitigate adverse COVID-19 outcomes.

The overwhelming use of TNF-α inhibitors (88.5%) in the study cohort reflects their central role in axSpA management. In contrast, the remaining 11.5% of patients were treated with IL-17 inhibitors, which mirrors the relatively recent approval and reimbursement of this therapeutic option in Romania since 2017 for axSpA patients. The average of nearly two biological therapy changes prior to COVID-19 diagnosis highlights the challenges in achieving sustained disease control in axSpA, particularly because there is an insufficient variety of molecular markers available to effectively predict patients’ responses to treatment, a stringent problem for a myriad of ailments [96]. Whether these therapies influence COVID-19 outcomes remains a pertinent question; the current literature suggests that TNF-α inhibitors may reduce the risk of severe outcomes by modulating hyperinflammatory states, a hypothesis that aligns with the cytokine-driven pathogenesis of both axSpA and severe COVID-19 [6,31,46,97,98,99]. In the study cohort, TNF-α inhibitors were used by 25.3% of patients with serious SARS-CoV-2 infection, while IL-17 inhibitors were used by 23.8%. Our study confirms that TNF-α inhibitors and IL-17 inhibitors did not significantly impact COVID-19 severity (*p* = 0.882), aligning with global findings [6,31,97,98,99]. However, our real-world dataset offers valuable insights into treatment modifications during infection, including temporary suspension and therapy-switching patterns in a national cohort. These findings highlight the importance of individualized approaches in managing immunosuppressive therapies during infectious outbreaks.

### Limitations

The major limitations of our study that could have influenced our results stem from the potential underrepresentation of the actual number of COVID-19 cases, as well as missing information, as reporting this infection in the RRBR was not mandatory (an inherent challenge in registry-based research). Our cohort is relatively small compared to larger population datasets, and conclusions should be interpreted with caution. This could have affected our results. Global efforts have been made for proactive infection surveillance, as recorded in international registries such as the Global Rheumatology Alliance (GRA) [100], the EULAR COVID-19 Rheumatology Registry [101] in collaboration with other National SARS-CoV-2 Registries (France, Germany [102], Greece [103], Italy, Portugal, and Sweden [104]), and the Brazilian Registry of Rheumatic Diseases and COVID-19 (ReumaCoV Brasil) [105]. Unlike these global registries, the RRBR does not systematically collect infection-related data, as its primary objective remains treatment monitoring rather than tracking pandemic-related outcomes. While some Romanian rheumatologists contributed data to the EULAR COVID-19 Registry, there is currently no dedicated national COVID-19 database for IMIDs in Romania.

Ideally, future registry development should consider a hybrid model, combining the structured, treatment-focused design of the RRBR with the pandemic-responsive frameworks of the GRA and EULAR. Such an approach would facilitate the real-time collection of infection-related data, improving preparedness for future health crises affecting rheumatic patients.

Furthermore, due to data privacy protection, we did not have a cross-checking system with healthcare records, lacking data about the vaccine type or timing, body mass index, axSpA activity scores, the infection treatment, or detailed comorbidities, especially pulmonary diseases and the specific restrictive lung patterns observed in these patients attributable to the structural alterations in the rib cage and thoracic spine. Such factors could further elucidate the severity of infection in certain cases.

## 5. Conclusions

This registry-based study enhances the understanding of axSpA in the context of COVID-19, highlighting the intricate relationship between chronic inflammatory diseases and infectious pathogens.

This study highlights the need for tailored management strategies for axSpA patients during infectious disease outbreaks. The observed patterns underscore the importance of multidisciplinary care and proactive measures to address potential complications, especially the importance of targeting older, less-educated individuals with comorbidities like HTN and osteoporosis. Lessons learned from the COVID-19 pandemic can inform future public health responses to protect vulnerable groups effectively.

Future research should explore the immunological interplay between axSpA and COVID-19 or other prevalent respiratory viruses, including the role of HLA-B27 and biological therapies in modulating disease severity. Long-term follow-up studies that include bigger cohorts and comparing data with other IMIDs and healthy controls are also essential to evaluate the pandemic’s impact on axSpA disease activity, treatment adherence, and patient outcomes, an endeavor that we are planning to fulfill. Additionally, as mentioned above, a dedicated Romanian Registry for comprehensive infection-related data collection is essential for future pandemics.

## Figures and Tables

**Table 1:** Demographic characteristics of patients in the cohort.

Characteristic	Total (n = 183)	Females (n = 65)	Males (n = 118)
**Age at COVID-19 Diagnosis (Years ± SD)**	48.19 ± 12.26	48.32 ± 14.12	48.12 ± 11.17
**Residence (n, %)**			
Urban	138 (75.4%)	49 (35.5%)	89 (64.5%)
Rural	45 (24.6%)	16 (35.5%)	29 (64.5%)
**Education (n, %)**			
Elementary + High School	96 (52.5%)	37 (38.5%)	59 (61.5%)
Higher Education	87 (47.5%)	28 (32.2%)	59 (67.8%)
**Smoking status (n, %)**			
Smokers	27 (14.8%)	8 (29.6%)	19 (70.4%)
Non-Smokers	156 (85.2%)	57 (36.5%)	99 (63.5%)

**Table 2:** The univariate binomial logistic regression analysis for variables of concern.

Predictor	N	Serious COVID-19 (N)	OR (95% CI) ^1^	*p*-Value
Sex				
F	65	20	—	
M	118	26	0.64 (0.32 to 1.27)	0.194
**Age**	**183**	**46**	**1.04 (1.01 to 1.07)**	**0.003**
**Age Category**				
**<52.5 years old**	**121**	**21**	—	
**≥52.5 years old**	**62**	**25**	**3.22 (1.62 to 6.49)**	**<0.001**
Current residence				
Rural	45	15	—	
Urban	138	31	0.58 (0.28 to 1.23)	0.147
**Level of Education**				
**Elementary + High School**	**96**	**32**	—	
**Higher Education**	**87**	**14**	**0.38 (0.18 to 0.76)**	**0.008**
Smoking status				
No	156	38	—	
Yes	27	8	1.31 (0.50 to 3.14)	0.561
Pharmacological treatment				
MTX				
No	175	45		
Yes	8	1	0.41 (0.05 to 3.44)	0.414
SSZ				
No	170	41	—	
Yes	13	5	1.97 (0.57 to 6.23)	0.258
Biological therapy				
IL-17 inhibitors	21	5	—	
TNF-α inhibitors	162	41	1.08 (0.40 to 3.48)	0.882
Extra-musculoskeletal involvement				
Ocular				
No	129	36	—	
Yes	54	10	0.59 (0.26 to 1.25)	0.185
COVID-19 vaccination				
No	95	26	—	
Yes	88	20	0.78 (0.40 to 1.52)	0.470
Comorbidities				
**HTN**				
**No**	**125**	**24**	—	
**Yes**	**58**	**22**	**2.57 (1.29 to 5.16)**	**0.007**
Ischemic heart disease				
No	167	41	—	
Yes	16	5	1.40 (0.42 to 4.09)	0.557
Chronic cardiac failure				
No	177	44	—	
Yes	6	2	1.51 (0.20 to 8.02)	0.64
Diabetes mellitus				
No	167	42	—	
Yes	16	4	0.99 (0.27 to 3.02)	0.989
**Osteoporosis**				
**No**	**173**	**41**	—	
**Yes**	**10**	**5**	**3.22 (0.86 to 12.1)**	**0.075**
HLA-B27				
No	65	20	—	
Yes	118	26	0.64 (0.32 to 1.27)	0.194

^1^ OR = Odds Ratio, CI = Confidence Interval.

**Table 3:** The multiple univariate binomial logistic regression analysis for variables of interest. OR = Odds Ratio, CI = Confidence Interval, VIF = Variance Inflation Factor.

Predictor	N	Serious COVID-19 (N)	OR (95% CI)	*p*-Value	VIF
**Age**	183	46	0.98 (0.93 to 1.04)	0.58	4.4
**Age Category**					3.7
<52.5 years old	121	21	-	-	-
≥52.5 years old	62	25	2.91 (0.76 to 11.6)	0.12	-
**Education**					
Elementary + High School	96	32	-	-	-
Higher	87	14	0.54 (0.24 to 1.19)		-
**Arterial Hypertension**					
No	125	24	-	-	-
Yes	58	22	1.62 (0.70 to 3.75)	0.26	-
**Osteoporosis**					
No	173	41	-	-	-
Yes	10	5	2.27 (0.56 to 9.15)	0.24	-

**Table 4:** The final predictive model for variables of interest.

Predictor	N	Serious COVID-19 (N)	OR (95% CI)	*p*-Value	VIF
**Age Category**					1.1
<52.5 years old	121	21	-	-	-
≥52.5 years old	62	25	2.64 (1.28 to 5.48)	0.009	-
**Education**					1.1
Elementary + High School	96	32	-	-	-
Higher Education	87	14	0.51 (0.23 to 1.06)	0.075	-

## Data Availability

The data presented in this study are available at the request of the corresponding author for legal and ethical reasons.

## Supplementary Materials

The following supporting information can be downloaded at https://www.mdpi.com/article/10.3390/medicina61030411/s1. Table S1: STROBE Statement—Checklist of items that should be included in reports of cohort studies.

## Abbreviation

ACE-2	Angiotensin-Converting Enzyme 2
ANG II	Angiotensin II
ARDS	Acute Respiratory Distress Syndrome
AUC	Area Under the Curve
axSpA	Axial Spondyloarthritis
bDMARDs	Biologic Disease-Modifying Antirheumatic Drugs
BMD	Bone Mineral Density
BP	Blood pressure
CAD	Coronary Artery Disease
CD	Cluster of Differentiation
CHF	Congestive Heart Failure
CI	Confidence Interval
COVID-19	Coronavirus Disease-2019
CRP	C-Reactive Protein
csDMARDs	Conventional Synthetic Disease-Modifying Antirheumatic Drugs
CT	computed tomography
F	Female
GCP	Good Clinical Practice
GDPR	General Data Protection Regulation
GRA	Global Rheumatology Alliance
HIV	Human Immunodeficiency Virus
HLA-B27	Human Leukocyte Antigen-B27
HTN	Arterial hypertension
IBD	Inflammatory Bowel Disease
ICU	Intensive Care Unit
IgA	Immunoglobulin A
IL	Interleukin
IL-17	Interleukin 17
IMIDs	Immune-Mediated Inflammatory Diseases
JAKi	Janus Kinase Inhibitor
M	Male
MRI	Magnetic Resonance Imaging
MTX	Methotrexate
N	Number
NSAIDs	Nonsteroidal Anti-inflammatory Drugs
OR	Odds Ratio
PAD	Peripheral Artery Disease
PsA	Psoriatic Arthritis
PUD	Peptic ulcer disease
QoL	Quality of Life
RAAS	Renin–Angiotensin–Aldosterone System
ROC	Receiver Operating Characteristic
RRBR	Romanian Registry of Rheumatic Diseases
RT-PCR	Reverse Transcription Polymerase Chain Reaction
SARS-CoV-2	Severe Acute Respiratory Syndrome Coronavirus 2
SD	Standard Deviation
SpO2	Peripheral Capillary Oxygen Saturation
SSZ	Sulfasalazine
STROBE	Strengthening the Reporting of Observational studies in Epidemiology
T_c_	Cytotoxic T-cell
TGF-β	Transforming Growth Factor β
TNF-α	Tumor Necrosis Factor-Alpha
tsDMARDs	Targeted Synthetic Disease-Modifying Antirheumatic Drugs
VIF	Variance Inflation Factor
WHO	World Health Organization
