# Supplementary material for: Predictive Factors for COVID-19 Severity in Patients with Axial Spondyloarthritis: Real-World Data from the Romanian Registry of Rheumatic Diseases
Source: Medicina (Kaunas). 2025 Feb 26;61(3):411. doi: 10.3390/medicina61030411 (PMC11943843; doi:10.3390/medicina61030411)
Supplement: Supplementary file 1 [file medicina-61-00411-s001.zip › medicina-3446971-supplementary.pdf]

Supplementary Table S1 - STROBE Statement—Checklist of items that should be included in reports of cohort *studies* – corresponding to the original article: "Predictive Factors for COVID-19 Severity in Patients with Axial Spondyloarthritis: Real-World Data from the Romanian Registry of Rheumatic Diseases"

	Item No	Recommendation
**Title and abstract**	1	(*a*) Indicate the study's design with a commonly used term in the title or the abstract<br>**Comment**: The design ("retrospective observational cohort study") is stated in the abstract and methods section.<br>**Abstract (Page 1, Line 38)**
		(*b*) Provide in the abstract an informative and balanced summary of what was done and what was found<br>**Comment**: The abstract includes the objectives, study design, key findings (e.g., predictors of severe COVID-19), and conclusions.<br>Refer to **Abstract (Page 1, Lines 38-55)**
**Introduction**		
Background/rationale	2	Explain the scientific background and rationale for the investigation being reported<br>**Comment**: The introduction explains the risks posed by COVID-19 in patients with immune-mediated diseases, specifically axial spondyloarthritis (axSpA). It also highlights gaps in understanding predictive factors for severe COVID-19 outcomes in this population.<br>Refer to **Introduction (Pages 2 and 3, Lines 65–149)**.
Objectives	3	State specific objectives, including any prespecified hypotheses<br>**Comment**: The main objective is to identify predictive factors for severe COVID-19 outcomes. A secondary objective involves performing a descriptive analysis of the cohort's clinical and demographic characteristics.<br>Refer to **Introduction (Page 3, Lines 116–153)**.
**Methods**		
Study design	4	Present key elements of study design early in the paper<br>**Comment**: The design is described as a retrospective observational cohort study, leveraging the Romanian Registry of Rheumatic Diseases.<br>Refer to **Material and Methods (Page 3, Lines 155-159)**
Setting	5	Describe the setting, locations, and relevant dates, including periods of recruitment, exposure, follow-up, and data collection<br>**Comment**: Data were collected from the Romanian Registry of Rheumatic Diseases (RRBR) between March 1, 2020, and December 31, 2023.<br>Refer to **Materials and Methods (Page 4, Lines 168–202)**.
Participants	6	(*a*) Give the eligibility criteria, and the sources and methods of selection of participants. Describe methods of follow-up<br>**Comment**: Inclusion criteria: Patients diagnosed with axSpA, confirmed COVID-19 (via RT-PCR or Rapid Antigen Test), and receiving biological treatments.<br>Exclusion criteria: Patients under 18 years old, those unable to consent, or with unconfirmed COVID-19 diagnoses.<br>Refer to **Materials and Methods (Page 4, Lines 177–202)**.
		(*b*) For matched studies, give matching criteria and number of exposed and unexposed<br>**Comment**: N/A
Variables	7	Clearly define all outcomes, exposures, predictors, potential confounders, and effect modifiers. Give diagnostic criteria, if applicable<br>**Comment**: Outcomes, exposures, and potential confounders are defined. Predictors

		include age, sex, HLA-B27 status, comorbidities (e.g., hypertension, osteoporosis), and treatment types.<br>Refer to **Materials and Methods (Pages 4 and 5, Lines 183–224)**.
Data sources/ measurement	8*	For each variable of interest, give sources of data and details of methods of assessment (measurement). Describe comparability of assessment methods if there is more than one group<br>**Comment**: Data were collected from the RRBR, anonymized, and harmonized before analysis. COVID-19 diagnosis was confirmed using validated methods (RT-PCR or Rapid Antigen Test).<br>Refer to **Materials and Methods (Pages 3-5, Lines 162–224)**.
Bias	9	Describe any efforts to address potential sources of bias<br>**Comment**: Potential biases were addressed by excluding incomplete data and anonymizing datasets. Variables with multicollinearity were excluded using Variance Inflation Factors (VIF).<br>Refer to **Materials and Methods (Page 3-5, Lines 162–224)**.
Study size	10	Explain how the study size was arrived at<br>**Comment**: The study size included 183 COVID-19 cases from 5,786 patients with axSpA. No formal power calculation was reported.<br>Refer to **Materials and Methods (Page 3, Lines 155-156)**.
Quantitative variables	11	Explain how quantitative variables were handled in the analyses. If applicable, describe which groupings were chosen and why<br>**Comment**: Age was grouped using ROC analysis to define a cutoff of 52.5 years as a predictor for severe outcomes. Other quantitative variables (e.g., smoking duration) are handled similarly.<br>Refer to **Materials and Methods (Pages 3-4, Lines 205-244)**.
Statistical methods	12	(*a*) Describe all statistical methods, including those used to control for confounding<br>**Comment**: Univariate and multivariate logistic regression were applied to assess predictors. VIFs addressed multicollinearity, and backward selection refined the predictive model.<br>Refer to **Materials and Methods (Pages 3-4, Lines 205-244)**.
		(*b*) Describe any methods used to examine subgroups and interactions
		(*c*) Explain how missing data were addressed
		(*d*) If applicable, explain how loss to follow-up was addressed
		(*e*) Describe any sensitivity analyses
**Results**		
Participants	13*	(a) Report numbers of individuals at each stage of study—eg numbers potentially eligible, examined for eligibility, confirmed eligible, included in the study, completing follow-up, and analysed<br>**Comment**: A flow of participants is described: 5,786 axSpA patients in the Registry, with 183 diagnosed with COVID-19. Gender, residence, education, and smoking habits are summarized.<br>Refer to **Results (Pages 5-6, Lines 246—272)**.
		(b) Give reasons for non-participation at each stage
		(c) Consider use of a flow diagram
Descriptive data	14*	(a) Give characteristics of study participants (eg demographic, clinical, social) and information on exposures and potential confounders<br>**Comment**: Demographics (e.g., age, residence), axSpA characteristics (e.g., disease duration, HLA-B27 status, specific treatments), and comorbidities are detailed.<br>Refer to **Results (Page 5-8, Lines 246–351)**.

		(b) Indicate number of participants with missing data for each variable of interest
		(c) Summarise follow-up time (eg, average and total amount)
Outcome data	15*	Report numbers of outcome events or summary measures over time<br>**Comment**: Serious COVID-19 was defined as requiring oxygen supplementation (25.1% of cases). Predictors for serious outcomes included age ≥52.5 years (OR 2.64, 95% CI: 1.28-5.48, p=0.009), arterial hypertension (OR 2.57, 95% CI: 1.29-5.16, p=0.007), and marginally non-significant – osteoporosis (OR 3.22, 95% CI: 0.86-12.1, p = 0.07). Higher education emerged as a protective factor (OR 0.38, 95% CI: 0.18-0.76, p=0.008).<br>Refer to **Results (Pages 9–12, Lines 403-489)**.
Main results	16	(*a*) Give unadjusted estimates and, if applicable, confounder-adjusted estimates and their precision (eg, 95% confidence interval). Make clear which confounders were adjusted for and why they were included<br>**Comment**: Adjusted odds ratios for serious COVID-19 predictors (e.g., age ≥52.5, hypertension) are provided with confidence intervals.<br>Refer to **Results (Pages 9–12, Lines 411–489)**.
		(*b*) Report category boundaries when continuous variables were categorized
		(*c*) If relevant, consider translating estimates of relative risk into absolute risk for a meaningful time period
Other analyses	17	Report other analyses done—eg analyses of subgroups and interactions, and sensitivity analyses<br>**Comment**: Subgroup analyses compared HLA-B27-positive and negative patients, as well as biological therapy types (TNF-α vs. IL-17 inhibitors) and csDMARDs.<br>Refer to **Results (Page 11, Lines 462-466 and 442-450)**.
**Discussion**		
Key results	18	Summarise key results with reference to study objectives<br>**Comment**: The study identified older age, hypertension, and osteoporosis as predictors of serious COVID-19, while higher education was protective.<br>Refer to **Discussion (Pages 13-15, Lines 512–546, 580-619)**.
Limitations	19	Discuss limitations of the study, taking into account sources of potential bias or imprecision. Discuss both direction and magnitude of any potential bias<br>**Comment**: Missing data, underrepresentation of certain comorbidities, and reliance on registry data are acknowledged as limitations.<br>Refer to **Discussion (Page 16, Lines 653–660)**.
Interpretation	20	Give a cautious overall interpretation of results considering objectives, limitations, multiplicity of analyses, results from similar studies, and other relevant evidence<br>**Comment**: The study emphasizes age, comorbidities, and education level as critical factors. It calls for tailored management of vulnerable populations during pandemics.<br>Refer to **Discussion and Conclusions (Pages 15-16, Lines 646-689)**.
Generalisability	21	Discuss the generalisability (external validity) of the study results<br>Results are relevant for axSpA populations in similar settings but may not generalize to broader or untreated cohorts.<br>Refer to **Discussion and Conclusions (Pages 15-16, Lines 646-689)**.
**Other information**		
Funding	22	Give the source of funding and the role of the funders for the present study and, if applicable, for the original study on which the present article is based<br>**Comment**: This research received no external funding.<br>Refer to **Funding (Page 17, Line 759)**

*Give information separately for exposed and unexposed groups.

Note: An Explanation and Elaboration article discusses each checklist item and gives methodological background and published examples of transparent reporting. The STROBE checklist is best used in conjunction with this article (freely available on the Web sites of PLoS Medicine at http://www.plosmedicine.org/, Annals of Internal Medicine at http://www.annals.org/, and Epidemiology at http://www.epidem.com/). Information on the STROBE Initiative is available at http://www.strobe-statement.org.
